# Enhanced HLA stability of a heteroclitic WT1 vaccine exacts a kinetic penalty in recognition by a natural TCR

**DOI:** 10.1016/j.jbc.2026.113373

**Published:** 2026-07-28

**Authors:** Min Yang, Qinglin Wei, Jiajia Wu, Peiluan Zhong, Xiaopeng Zhu, Pengcheng Wei

**Affiliations:** Guangxi Key Laboratory of Special Biomedicine, School of Medicine, Guangxi University, Nanning, China

**Keywords:** vaccine design, immunotherapy, TCR-T, Wilms’ tumor 1, crystal structure

## Abstract

Wilms’ tumor 1 (WT1) is aberrantly overexpressed across hematologic and solid malignancies and ranks among top-priority antigens for T cell receptor (TCR)-based immunotherapy. Among WT1 epitopes, the HLA-A∗02:01-restricted nonamer RMFPNAPYL (WT1_126_-_134_; RL9) has advanced clinically, including with the naturally selected C4 TCR. Despite encouraging activity in early trials, the molecular basis of C4 recognition and its tolerance to vaccine-motivated anchor modifications remain unclear. Here we report high-resolution crystal structures of C4 bound to HLA-A∗02:01 presenting either the native RL9 peptide or the R1Y variant (YMFPNAPYL; YL9). C4 docks diagonally with a peptide-centric footprint biased toward central and C-terminal positions, contrasting with TCR-mimic antibodies that depend on N-terminal R1 contacts. Although R1Y preserves the peptide backbone, p1Y reorients upon TCR engagement, eliminating the E94 (CDRα3)-p1R salt bridges, remodeling the electrostatic landscape, and weakening interfacial hydrogen-bond/salt-bridge networks. Differential scanning fluorimetry showed that YL9-HLA-A∗02:01 is more thermally stable than RL9-HLA-A∗02:01 (*T*_m_ = 54.0 °C ± 0.21 °C *versus* 48.9 °C ± 0.25 °C). Surface plasmon resonance revealed micromolar binding of C4 to RL9-HLA-A∗02:01 (*K*_D_ = 13.8 ± 1.53 μM) but weak binding to YL9-HLA-A∗02:01 (*K*_D_ > 150 μM). Together, these structural and biophysical data clarify the recognition logic of a clinically validated WT1-specific TCR and reveal that an HLA-stabilizing R1Y modification can reduce recognition by C4, even while improving pHLA stability. These findings do not preclude YL9 from eliciting other cross-reactive TCR clonotypes, but highlight the need to evaluate both HLA stabilization and TCR-recognition fidelity during epitope selection and structure-informed TCR engineering in WT1-targeted immunotherapy.

Wilms’ tumor 1 (WT1) is a zinc-finger transcription factor aberrantly overexpressed in a variety of hematologic malignancies and solid tumors, including acute myeloid leukemia (AML), mesothelioma, and ovarian carcinoma (1). Owing to its limited expression in normal adult tissues and its oncogenic role in malignant transformation, WT1 was ranked a top-priority cancer antigen for immunotherapeutic targeting by the U.S. National Cancer Institute (2). Among WT1-derived epitopes, the HLA-A∗02:01-restricted nonamer RMFPNAPYL (WT1_126_–_134_; RL9) exhibits favorable presentation and robust immunogenicity and has been advanced in peptide vaccines, T-cell receptor (TCR)-engineered T cells (TCR-T), and TCR-mimic (TCRm) antibodies (3, 4, 5, 6, 7, 8).

Unlike chimeric antigen receptor (CAR)-T cells, which target surface antigens, TCR-based therapies access intracellular proteins *via* peptide-HLA (pHLA) presentation (9). Within WT1-directed TCR-T, the naturally selected C4 TCR, which is isolated from a HLA-A∗02:01^+^ healthy donor and chosen for high-avidity RL9 recognition, has shown compelling activity, including encouraging outcomes in a phase I trial following hematopoietic cell transplantation (3). Yet the molecular mechanism by which C4 engages its cognate pHLA remains undefined. In particular, we lack an atomic-level description of the C4 footprint and energetics on RL9, knowledge of its tolerance to clinically deployed epitope variants, and quantitative binding kinetics to benchmark antigen sensitivity, all of which are essential for rational TCR and vaccine design.

Anchor optimization and heteroclitic/mimotope substitutions are widely used to enhance HLA binding and T-cell priming, but it might yield mixed consequences for epitope fidelity and cross-recognition (10, 11, 12). Mechanistic studies, including work from our group, implicate pMHC electrostatic remodeling, shifts in TCR docking geometry, and redistribution of peptide contacts as key determinants of these outcomes (12, 13, 14, 15, 16). In the WT1 system, a particularly relevant variant substitutes the N-terminal Arginine (p1R) with Tyrosine (p1Y) in YMFPNAPYL (R1Y; “YL9”) to increase HLA-A∗02:01 stability and immunogenicity (1, 17, 18). YL9 has advanced into peptide-vaccine formulations (*e*.*g*., galinpepimut-S) with favorable safety and immunogenicity in early trials (17, 18, 19, 20, 21, 22), yet structural analyses suggest that R1Y can alter the pHLA electrostatic landscape and TCR-exposed surface (7). TCRm antibodies (ESK1, 11D06) and the affinity-enhanced α7β2 TCR rely heavily on p1R-mediated contacts, prompting the question of whether natural TCRs such as C4 display broader tolerance or alternative docking solutions (5, 6, 23). More broadly, “p1Y” substitutions have been deployed across multiple tumor-associated antigens—including hTERT, MUC1, and POTE—under the prevailing assumption that they enhance HLA-A2 binding while preserving T-cell specificity (11, 24, 25, 26, 27).

Accordingly, defining how natural TCRs accommodate, or fail to accommodate, R1Y is pivotal for rational peptide-vaccine design, providing mechanistic criteria to guide candidate selection and to assess whether p1Y-based immunogens are appropriate and likely to be clinically effective. Here, we present high-resolution structures of C4 bound to HLA-A∗02:01 presenting either native RL9 or the R1Y (YL9) variant, complemented by surface plasmon resonance (SPR) binding kinetics and differential scanning fluorimetry (DSF) stability measurements. These comparative structural and energetic analyses delineate the molecular logic of C4 recognition and inform strategies for epitope refinement, variant risk assessment, and structure-informed TCR engineering in WT1-targeted immunotherapy.

## Results

### Overall architecture of the C4 TCR–RL9–HLA-A∗02:01 complex

**Figure 1:**
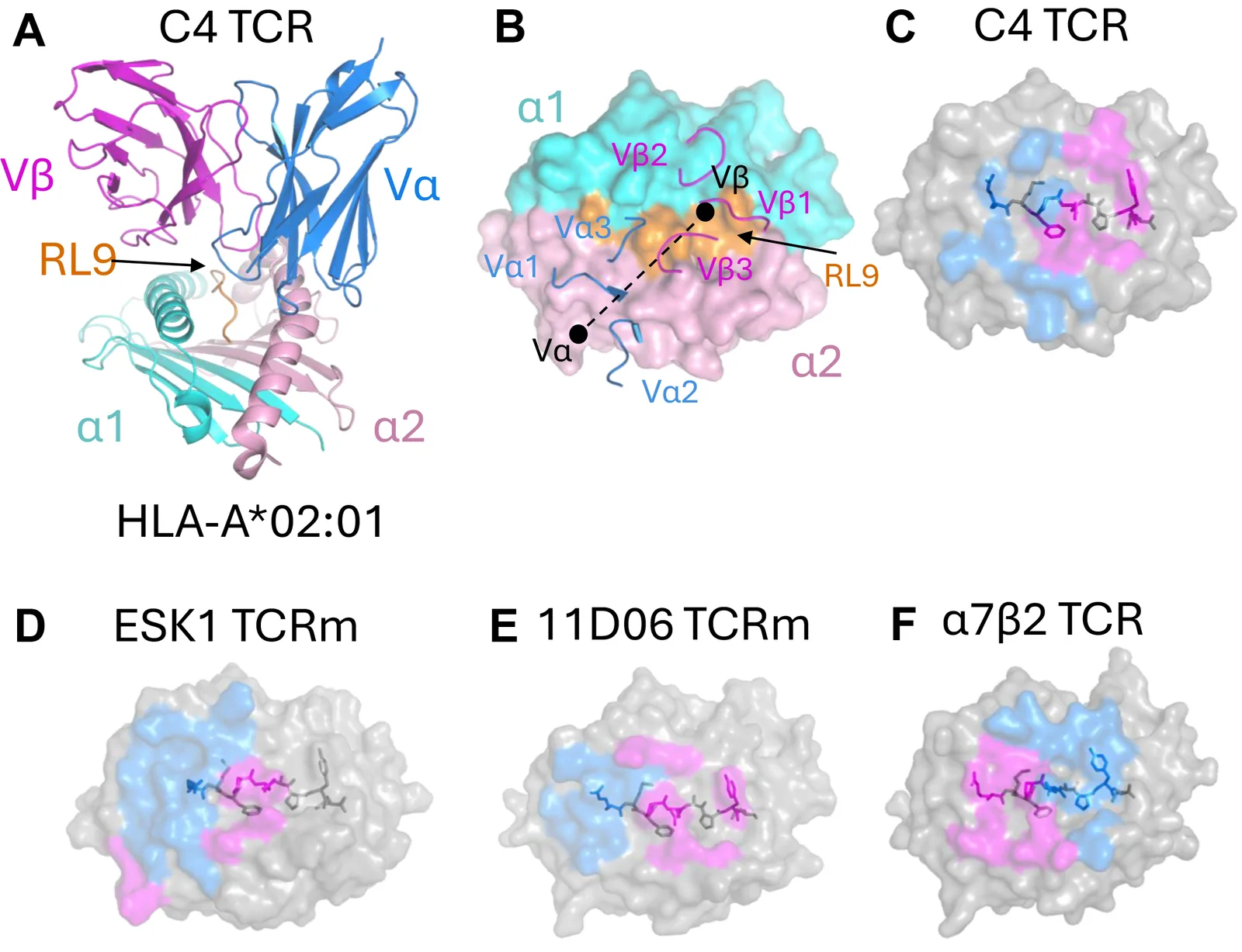
**Overall architecture and footprints of C4 TCR on RL9–HLA-A∗02:01.***A*, ternary complex shown in cartoon. TCR Vα (*marine*) and Vβ (*magenta*) dock diagonally (∼48°) over HLA-A∗02:01 α2 (*pink*) and α1 (*cyan*), respectively; the RL9 peptide (*orange*) sits in the groove. *B*, distribution of TCR CDR loops mapped on the pHLA surface; centers of mass for Vα and Vβ are indicated by *black dots*. (*C–F*) Footprints on RL9–HLA-A∗02:01 for C4, ESK1 (TCRm), 11D06 (TCRm), and the engineered α7β2 TCR. Vα footprints are rendered in *marine*; Vβ footprints in *magenta*. C4, 11D06, and α7β2 share a peptide-centric diagonal orientation, whereas ESK1 concentrates at the peptide N-terminus. Structural models were generated from the refined crystal structures, and crystallographic data-collection and refinement statistics are provided in Table 1.

To define how a natural TCR engages the WT1 epitope, we determined the 2.25 Å crystal structure of the C4 TCR–RL9–HLA-A∗02:01 ternary complex, with complete data-collection, processing, and refinement statistics provided in Table 1. C4 docks in a canonical diagonal orientation, crossing the peptide-binding cleft at ∼48°, with Vα positioned over the HLA α2 helix and Vβ over α1 (Fig. 1, *A* and *B*). The buried surface area at the C4-RL9-HLA-A∗02:01 interface is ∼828.3 Å^2^ (≈3.9% of the complex’s total solvent-accessible surface of ∼21,241.5 Å^2^). Visual inspection reveals tight packing at the peptide center and complementary ridges/grooves along the HLA helices, yielding a contiguous footprint without steric clashes near the CDR apices.

**Table 1:** Diffraction data collection and refinement statistics^a^

Data set	C4 TCR-HLA-A∗02:01-RL9	C4 TCR-HLA-A∗02:01-YL9
PDB Code	9WC2	9WC3
Data Collection		
Space group	P 2_1_ 2_1_ 2_1_	P 2_1_ 2_1_ 2_1_
Cell dimensions		
*a*, *b*, *c* (Å)	70.4, 79.0, 193.6	72.4, 79.1, 191.7
*α*, *β*, *γ* (°)	90, 90, 90	90, 90, 90
Resolution range (Å)	49.96–2.25 (2.32–2.25)	49.71–2.40 (2.49–2.40)
R_merge_^b^ (%)	12.7 (48.1)	14 (52.6)
Mean I/sigma (I)	11.7 (4.2)	6.8 (2.4)
CC (1/2)	0.995	0.976
Completeness (%)	98.3 (93.4)	96.6 (96.5)
Multiplicity	10.4 (9.6)	4.2 (3.7)
Refinement		
No.Reflections	51,097 (4755)	39,335 (3524)
Complexes per A.U.^c^	1	1
R_work_/R_free_ (%)	19.24 (22.69)/23.14 (31.93)	19.13 (24.23)/23.51 (29.92)
No. of atoms		
Protein	6617	6619
water	317	364
Average B factor	35.05	25.08
Protein	35.00	25.09
Water	36.12	24.97
R.m.s deviations		
RMS(bonds) (Å)	0.003	0.003
RMS(angles) (°)	0.66	0.62
Ramachandran plot		
Favored (%)	96.78	96.77
Allowed (%)	3.22	3.10
Disallowed (%)	0	0.12

a Data for outer resolution shell is shown in parentheses.

b Rmerge = ΣhklΣi |Ii(hkl) − ⟨I(hkl)⟩|/ΣhklΣi Ii(hkl), where Ii(hkl) is the intensity of the i-th observation of reflection hkl, and ⟨I(hkl)⟩ is the mean intensity of all observations of that reflection.

c A.U = asymmetric unit, the minimum portion of the crystal unit cell that can be used to complete the unit cells *via* the symmetry of the space group.

A footprint comparison with other RL9 binders highlights mode-of-recognition differences (Fig. 1, *C*–*F*), and the detailed contacts on peptides were presented in Table S1. C4 most closely resembles 11D06 (TCRm) and the engineered α7β2 TCR in its peptide-centric, diagonal docking and contact distributions across central/C-terminal positions. By contrast, ESK1 (TCRm) exhibits an N-terminal-biased footprint that concentrates on p1R/p4P, consistent with its sensitivity to alterations at these sites. Thus, even at the global level, C4 behaves like a physiologically selected TCR that prioritizes the peptide’s center while maintaining stabilizing interactions with both HLA helices.

Together, these features establish the baseline docking geometry and epitope presentation for subsequent residue-level analyses and variant comparisons.

### Mapping of interfacial contributions and roles of individual CDR loops for RL9 recognition

**Figure 2:**
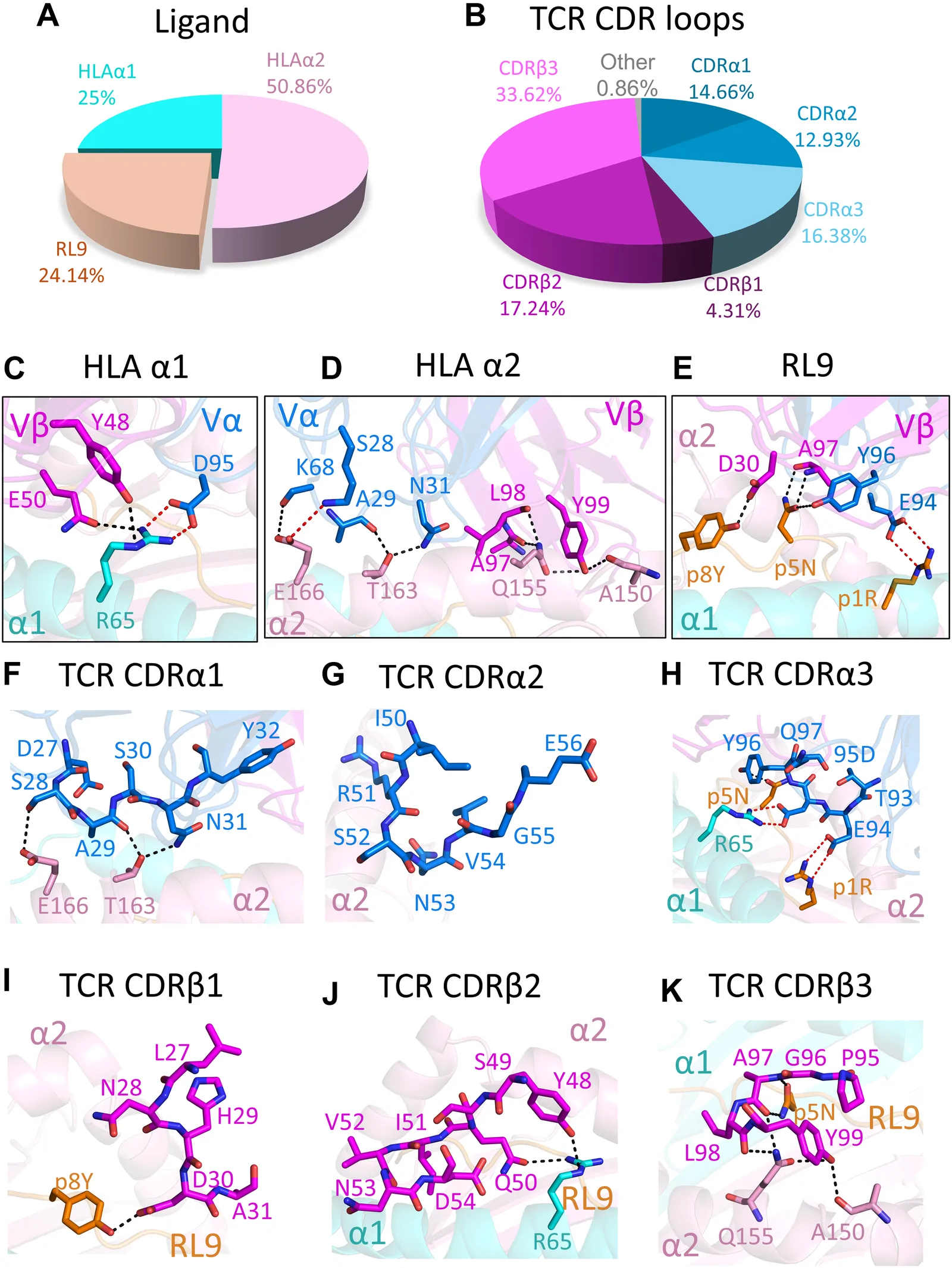
**Interaction networks underlying C4 TCR recognition of RL9–HLA-A∗02:01.***A*, Pie chart partitioning interfacial contacts attributed to the peptide (*orange*), HLA α1 (*cyan*), and HLA α2 (*pink*). *B*, Relative contributions of the six CDR loops to the interface (CDRα1–3 in *blues*; CDRβ1–3 in *magentas*), highlighting CDRβ3 as the dominant contributor. The percentages in (*A*) and (*B*) are based on contact counts, including H-bonds, SBs, and vdW contacts. *C–E*, key residues and contacts on α1, α2, and the peptide. Examples include R65 (α1) forming SBs (*red dash*)/H-bonds (*black dash*) with D95 (CDRα3) and Y48/Q50 (CDRβ2); along α2 (A150–E166), A150/Q155 H-bond to Y99/A97/L98 (CDRβ3), and T163/E166 contact A29/N31/K68 (α-chain framework); on the peptide, exposed positions p1R, p3F, p4P, p5N, p8Y engage E94 (CDRα3), A97 (CDRβ3), D30 (CDRα1), and CDRβ3 *via* SBs/H-bonds interactions as indicated. (*F–K*) Stick representations of CDRα1, CDRα2, CDRα3, CDRβ1, CDRβ2, and CDRβ3 in contact with RL9–HLA-A∗02:01. H-bonds and SBs are annotated. Residues are labeled with single-letter codes (*e*.*g*., E94, D95, Y96, R65, W167, E166).

To describe how C4 contacts RL9–HLA-A∗02:01, we assigned direct interatomic contacts, including hydrogen bonds (H-bonds), salt bridges (SBs), and van der Waals (vdW) contacts, and grouped them by the contacted peptide/HLA region or TCR CDR loop. The resulting percentages represent contact-count distributions rather than buried surface area or energetic contributions. On the pHLA side, approximately 50.86% of assigned contacts involve the HLA α2 helix, 25.00% involve the HLA α1 helix, and 24.14% involve the peptide (Fig. 2*A*). On the TCR side, Vα- and Vβ-derived contacts account for approximately 44.83% and 55.17%, respectively, with CDRβ3 providing the largest single-loop share (∼33.62%); the remaining loops contribute CDRα1 ∼14.66%, CDRα2 ∼12.93%, CDRα3 ∼16.38%, CDRβ2 ∼17.24%, and CDRβ1 ∼4.31% (Fig. 2*B*). Specific contacts are detailed in Table S2.

At the residue level, α1-R65 is a dominant hot spot, forming two SBs with D95 (CDRα3) and H-bonds with Y48/Q50 (CDRβ2) (Fig. 2*C*). Along α2 (A150–E166), A150 and Q155 form H-bonds to Y99/A97/L98 (CDRβ3), and T163/E166 contact A29/N31/K68 on the α-chain framework (Fig. 2*D*). Among peptide contacts, the exposed positions p1R, p3F, p4P, p5N, p8Y are decoded by C4 as followed: p1R makes two SBs with E94 (CDRα3); p5N forms H-bonds with A97 (CDRβ3) and Y96 (CDRα3); p8Y forms one H-bonds with D30 (CDRβ1); and p3F/p4P engage in multiple vdWs contacts with CDRα3, CDRβ1, and CDRβ3 (Fig. 2*E* and Table S3).

Consistent with these residue-level networks, CDRα1 (D27–Y32) engages the C-terminal segment of α2 (A158–W167) through A29/N31 H-bonds, with an additional S28–E166 H-bond (Fig. 2*F*). CDRα2 packs against the central α2 (E154–E161) mainly *via* vdW interactions, and framework K68 forms a stabilizing SB with E166 (Fig. 2*G*). CDRα3 (T93–Q97) bridges the peptide and α1, featuring E94–p1R (two SBs), D95–R65 (SB), and Y96–p5N (H-bond) (Fig. 2*H*). On the β chain, CDRβ1 (L27–A31) sits over the peptide C-terminus to contact p8Y (one H-bond; four vdWs) (Fig. 2*I*); CDRβ2 (Y48–D54) engages R65 with two H-bonds (Fig. 2*J*); and CDRβ3 (P95–Y99) focuses on the peptide center and the N-terminal portion of α2, forming multiple H-bonds to p5N and α2 (Fig. 2*K*). Together these data explain why α1 is approached predominantly by CDRα3/CDRβ2, α2 by CDRα1/α2/CDRβ3, and why the exposed peptide positions (p1, p3–5, p8) are read chiefly by CDRα3, CDRβ1, and CDRβ3, with the remaining residues buried in the HLA groove.

### Molecular basis of C4 TCR recognition of YL9 presented by HLA-A∗02:01

**Figure 3:**
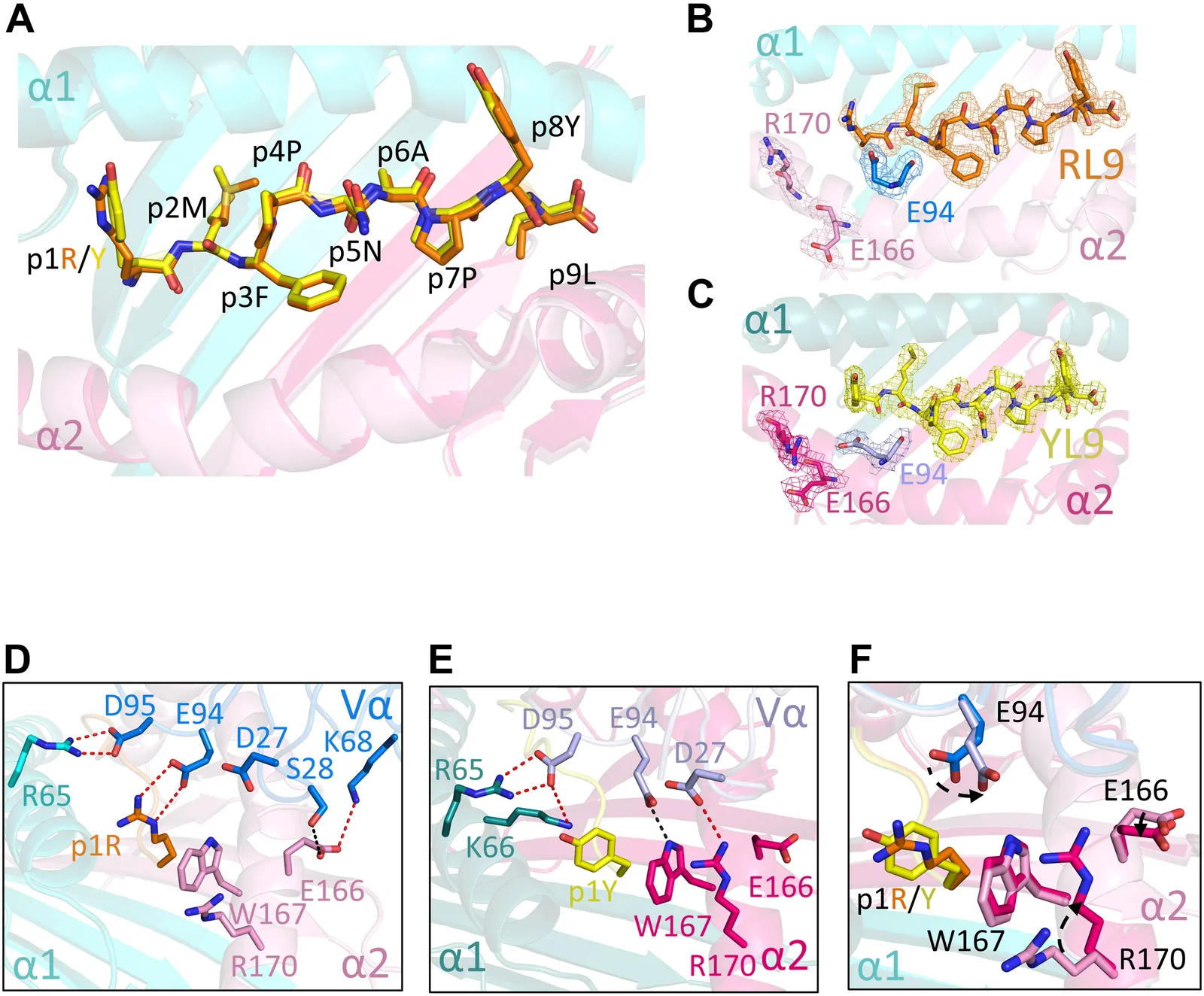
**Comparison of C4 TCR recognition of RL9 *versus* R1Y (YL9).***A*, overlay of RL9 (*orange*) and YL9 (*yellow*) peptides in the HLA-A∗02:01 groove, illustrating conserved backbone conformation and docking geometry under C4 engagement. (*B* and *C*) 2mFo–DFc electron-density maps, contoured at 1.0 σ, supporting the refined models of the RL9 and YL9 peptides and nearby interfacial residues in the C4 TCR–RL9–HLA-A∗02:01 and C4 TCR–YL9–HLA-A∗02:01 complexes, respectively. The peptide backbones are largely conserved, whereas the refined models show local side-chain differences, including a modest difference at p2M and a more prominent change at the p1R/p1Y position. Color scheme: RL9 complex—Vα (*marine*), Vβ (*magenta*), HLA α1 (*cyan*), HLA α2 (*pink*); YL9 complex—Vα (*light-blue*), Vβ (*violet*), α1 (*deep-teal*), α2 (*hot-pink*). *D*, p1-centered interaction network in the RL9 complex, highlighting two SBs between p1R and E94 (CDRα3) and contacts involving HLA R65. *E*, p1-centered interaction network in the YL9 complex, showing loss of the p1R–E94 (CDRα3) SBs and replacement by primarily vdW contacts involving p1Y. *F*, local rearrangements induced by the R1Y substitution, involving E94 (CDRα3), HLA W167/E166/R170, and TCRα D27/S28/K68. Compared with YL9, RL9 forms a more extensive direct polar-contact network with C4 at the peptide N terminus.

To assess tolerance to a clinically used anchor modification, we determined the 2.4 Å crystal structure of C4 TCR–YL9–HLA-A∗02:01, with complete data-collection, processing, and refinement statistics provided in Table 1. Structural overlay showed that the peptide backbones and HLA groove conformations are largely conserved between the RL9 and YL9 complexes (Fig. 3*A*). The refined models, supported by well-defined 2mFo–DFc electron-density maps, showed local side-chain differences, including a modest reorientation of p2M and a more pronounced change at the p1R/p1Y position (Fig. 3, *B* and *C*). The p2M side chain remains accommodated within the HLA groove, whereas the most prominent TCR-facing difference occurs at the peptide N terminus, where p1R is replaced by p1Y.

Comparison of direct TCR–pHLA contacts showed that the R1Y substitution neutralizes the p1 microenvironment and rewires the local contact network without substantially perturbing the global docking geometry (Tables S2–S5). In the RL9 complex, p1R forms two SBs with E94 in CDRα3, providing direct TCR–peptide electrostatic contacts. In the YL9 complex, p1Y cannot maintain these SBs and instead makes mainly vdW contacts (Fig. 3, *D* and *E* and Table S5). Concomitantly, E94 reorients toward HLA W167, D27 forms an SB with HLA R170, and E166 shifts toward R170, altering contacts involving S28/K68 (Fig. 3*F*). Thus, RL9 supports a more extensive direct TCR–peptide polar-contact network, whereas YL9 preserves the overall complex architecture but weakens the local electrostatic interactions that stabilize C4 recognition.

### Enhanced pHLA stability and altered binding kinetics define C4 TCR recognition of a p1-modified WT1 epitope

By DSF, both WT1-derived peptide–HLA complexes exhibited single cooperative unfolding transitions, consistent with homogeneous preparations. The R1Y (YL9)–HLA-A∗02:01 complex was markedly more stable, with a melting temperature (*T*_m_) of 54.0 °C ± 0.21 °C compared to 48.9 °C ± 0.25 °C for RL9–HLA-A∗02:01, reflecting enhanced stabilization conferred by the p1 anchor substitution (Fig. S1 and Table S6).

**Figure 4:**
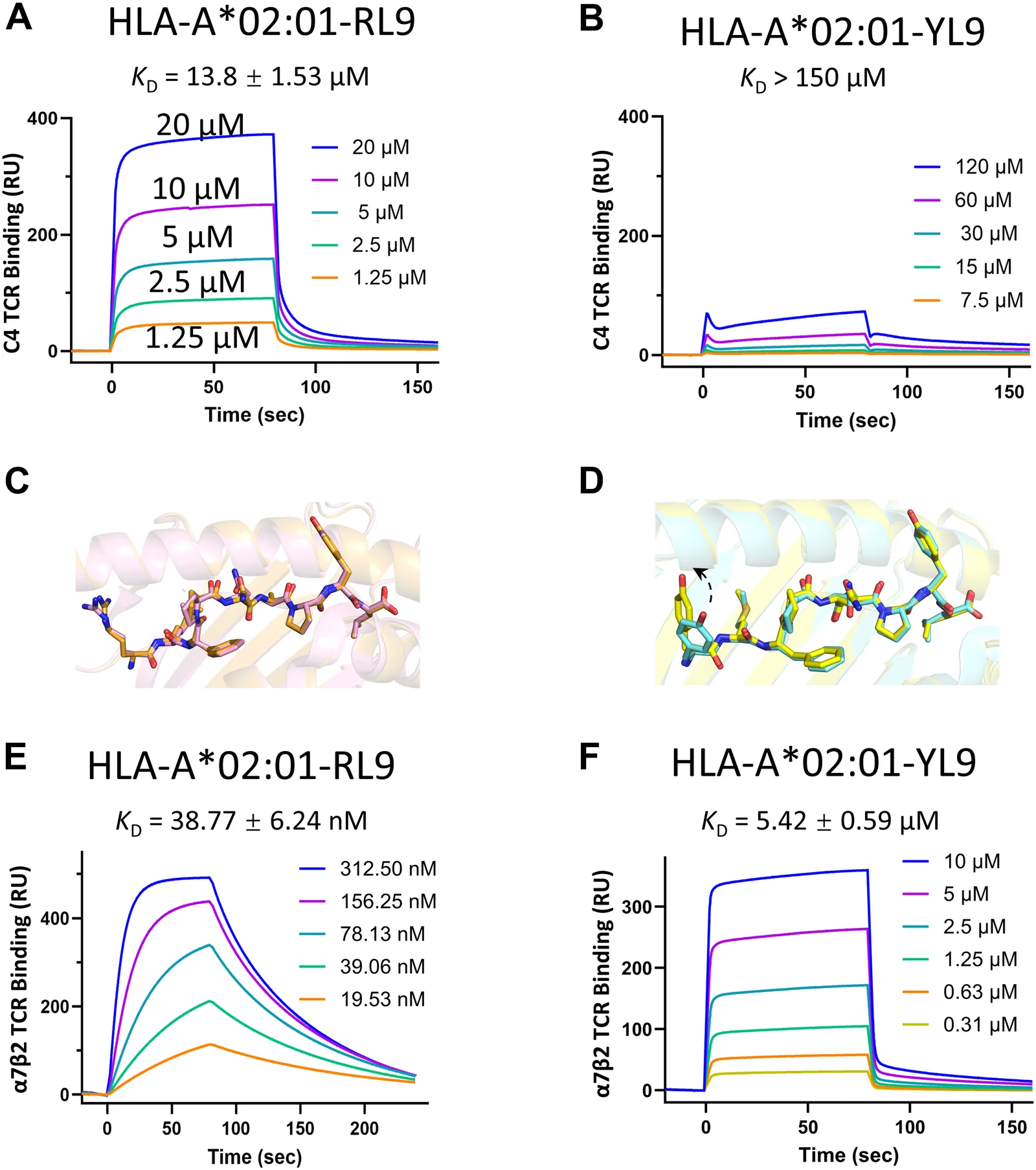
**SPR analysis of C4 and α7β2 TCR binding to HLA-A∗02:01 presenting RL9 or YL9, and peptide conformational changes upon TCR engagement.***A*, SPR sensorgrams for RL9–HLA-A∗02:01 injected over immobilized C4 TCR at serial concentrations. The C4–RL9 interaction showed biphasic association/dissociation behavior and was fitted using a two-state reaction model, yielding a *K*_D_ of 13.8 ± 1.53 μM. Model-dependent kinetic parameters are summarized in Table S7. *B*, SPR sensorgrams for YL9–HLA-A∗02:01 injected over immobilized C4 TCR. C4 binding to YL9–HLA-A∗02:01 was weak and did not support reliable kinetic fitting or precise *K*_D_ determination; therefore, this interaction is reported conservatively as *K*_D_ > 150 μM. *C*, structural overlay of unliganded RL9–HLA-A∗02:01 (*pink*; PDB 3HPJ) with the C4 TCR–RL9–HLA-A∗02:01 complex (*orange*). *D*, structural overlay of unliganded YL9–HLA-A∗02:01 (*cyan*; PDB 3MYJ) with the C4 TCR–YL9–HLA-A∗02:01 complex (*yellow*), highlighting the local displacement of p1Y upon TCR engagement. *E*, SPR sensorgrams for RL9–HLA-A∗02:01 injected over immobilized α7β2 TCR at serial concentrations. The α7β2–RL9 interaction was fitted using a 1:1 Langmuir binding model, yielding a *K*_D_ of 38.77 ± 6.24 nM. Kinetic parameters are summarized in Table S8. *F*, SPR sensorgrams for YL9–HLA-A∗02:01 injected over immobilized α7β2 TCR at serial concentrations. The α7β2–YL9 interaction showed biphasic association/dissociation behavior and was fitted using a two-state reaction model, yielding a *K*_D_ of 5.42 ± 0.59 μM. Model-dependent kinetic parameters are summarized in Table S8. *K*_D_ values are reported as mean ± S.D. from three independent replicate SPR measurements where reliable fitting was possible.

SPR further underscored the contrasting effects of this modification. C4 TCR bound native RL9–HLA-A∗02:01 with a *K*_D_ of 13.8 ± 1.53 μM based on three independent replicate measurements (Fig. 4*A* and Table S7). Because the C4–RL9 sensorgrams showed biphasic association/dissociation behavior, they were fitted using a two-state reaction model with the model-dependent kinetic parameters summarized in Table S7. In contrast, C4 binding to the p1-modified YL9–HLA-A∗02:01 was substantially weaker in replicate measurements and did not support reliable kinetic fitting or precise *K*_D_ determination; therefore, this interaction is reported conservatively as *K*_D_ > 150 μM (Fig. 4*B*). Meanwhile, from the structure comparison of pHLA and C4-pHLA, p1R is slightly pressed downward (Fig. 4*C*) while p1Y undergoes a greater degree of downward pressure (Fig. 4*D*), which might explain the huge differences in C4 affinity. Thus, although the R1Y substitution increases overall pMHC stability, it substantially compromises TCR recognition. To validate the affinity differences caused by p1Y, we selected the engineered α7β2 TCR to assay its affinity with the two pHLAs (Fig. 4, *E* and *F*). The engineered α7β2 TCR bound RL9–HLA-A∗02:01 with a *K*_D_ of 38.77 ± 6.24 nM based on three independent replicate measurements and was fitted using a 1:1 Langmuir binding model (Fig. 4*E* and Table S8). In contrast, α7β2 binding to YL9–HLA-A∗02:01 was approximately 140-fold weaker, with a *K*_D_ of 5.42 ± 0.59 μM, and was fitted using a two-state reaction model because the sensorgrams showed biphasic association/dissociation behavior (Fig. 4*F* and Table S8). The corresponding kinetic parameters are summarized in Tables S7 and S8. Together, DSF and SPR define the biophysical consequences of the p1 substitution: enhanced intrinsic stability of the pMHC scaffold, but diminished TCR engagement due to kinetic penalties, thereby revealing the limits of C4 TCR tolerance for anchor-modified epitopes.

## Discussion

To our knowledge, this study provides the first structural and energetic dissection of how a naturally selected human TCR recognizes the clinically advanced WT1 epitope, RL9, and how this interaction is perturbed by an anchor-modified variant.

High-resolution structures establish that C4 docks diagonally across the HLA-A∗02:01–RL9 complex with a peptide-centric footprint. The interface is dominated by CDRβ3 and CDRα3 contributions, targeting central and C-terminal peptide residues while maintaining stabilizing contacts with both HLA helices. This architecture aligns with “physiological” docking modes of thymically selected TCRs (28) and contrasts with TCR-mimic antibodies such as ESK1 (6), which rely disproportionately on N-terminal contacts. The distribution of contacts suggests that natural WT1-specific TCRs balance peptide discrimination with HLA compatibility—an important consideration when evaluating engineered receptors for therapy.

A central insight is that C4, despite its focus on peptide center/C-terminus, is not permissive to the clinically used R1Y (p1Y) anchor modification. Although the peptide backbone is preserved, R1Y neutralizes the p1 microenvironment and disrupts the E94(CDRα3)–p1R salt-bridge pair, rewiring local contacts and altering the interfacial electrostatics. Biophysically, this yields substantially weaker binding: C4 binds native RL9–HLA-A∗02:01 with *K*_D_ = 13.8 ± 1.53 μM, whereas C4 binding to YL9–HLA-A∗02:01 is weak and does not support reliable kinetic fitting (*K*_D_ > 150 μM). Thus, the R1Y substitution substantially reduces C4 recognition. Thus, residues that appear distal to the dominant footprint can function as electrostatic keystones that stabilize docking geometry and set kinetic accessibility.

Although we did not directly measure C4-transduced T-cell activation by YL9 in this study, the reduced C4 affinity for YL9–HLA-A∗02:01 is expected, based on published TCR–pMHC functional studies, to decrease antigen sensitivity and/or raise the activation threshold for C4-bearing T cells. Previous studies have shown that TCR–pMHC binding parameters, including affinity, dissociation rate, association rate, and ligand density, can influence CD8+ T-cell responsiveness, cytokine production, cytotoxicity, polyfunctionality, proliferation, and antitumor activity (8, 29, 30, 31, 32). Thus, the markedly weaker C4–YL9 interaction would be predicted to require a higher density of YL9–HLA-A∗02:01 complexes for efficient C4-dependent activation.

Anchor optimization, especially p1Y substitution to enhance HLA-A2 stability, has been widely applied across multiple tumor-associated antigens, including WT1, hTERT, MUC1, and POTE (11, 24, 25, 26, 27). In the WT1 system, YL9 has been incorporated into peptide-vaccine formulations, including galinpepimut-S, and previous clinical and translational studies have reported favorable safety and immunogenicity profiles (17, 18, 19, 20, 21, 22). Although such substitutions can improve pHLA stability, our results show that enhanced stability does not necessarily guarantee preserved recognition by every therapeutically relevant native-epitope-specific TCR. In our system, YL9–HLA-A∗02:01 is thermally more stable than RL9–HLA-A∗02:01, yet C4 engagement is substantially reduced.

Importantly, this finding should not be interpreted as evidence that YL9 is intrinsically non-functional or unsuitable as a vaccine epitope. Nguyen *et al*. directly compared CD8+ T-cell responses to the native RL9 epitope RMFPNAPYL and the heteroclitic YL9 epitope YMFPNAPYL (8). They showed that YL9 vaccination can elicit functional cross-reactive RL9-specific CD8+ T-cell responses but also found that YL9-induced cross-reactive T cells and single RL9-reactive T cells display distinct TCRαβ signatures. Thus, the outcome of YL9 vaccination is likely repertoire dependent. Consistent with this broader functional context, earlier WT1 studies of the same RL9/YL9 epitope pair showed that the YL9 analog can improve HLA-A∗02:01 binding/stability and/or immunogenicity and can induce WT1-specific CD8+ T-cell responses capable of cross-recognizing native RL9 and lysing WT1-expressing leukemic cells (17, 18). In this context, our C4 data do not argue against the immunogenicity of YL9 *per se*; rather, they show that a therapeutically relevant native RL9-specific TCR can incur a substantial recognition penalty when engaging the YL9-modified surface. These observations support a TCR-aware model of heteroclitic vaccine design, in which enhanced HLA stability and antigen presentation should be balanced against preservation of the native tumor-epitope surface recognized by clinically relevant TCRs.

Our work examines one natural TCR against two peptide states with *in vitro* assays. Broader surveys of WT1-specific TCRs are needed to assess generality of p1 dependence, alongside repertoire studies of p1Y-elicited T cells to map cross-reactivity landscapes. *In vivo* tests will be required to connect structural/kinetic parameters—especially on-rate and dwell time—to persistence, phenotype, and efficacy of C4-engineered T cells. Exploring electrostatics-preserving anchor strategies that retain HLA stability and TCR fidelity represents a practical design direction.

By integrating crystallography, kinetics, and stability assays, we define why a clinically validated, naturally selected TCR recognizes WT1 with high specificity yet poorly tolerates a common anchor modification. Enhanced pMHC stability alone does not necessarily ensure preserved recognition by all therapeutically relevant TCRs, and its functional benefit is likely to depend on the TCR repertoire recruited by vaccination. These principles provide actionable guidance for authentic epitope selection, variant risk assessment, and structure-informed TCR engineering in WT1-targeted immunotherapy and in other peptide-based programs targeting antigens such as hTERT, MUC1, and POTE.

## Experimental procedures

### Protein expression and purification

Recombinant peptide-HLA-A∗02:01 (pHLA-A∗02:01) complexes were produced as described previously (33, 34). Briefly, the extracellular domain of the human *HLA*-*A∗02*:*01* heavy chain (residues 1–276) and human *β2-microglobulin* (*β2m*) were codon-optimized for *Escherichia coli* (*E*. *Coli*) expression and cloned into pET-28a(+) vectors. Both proteins were expressed in *E*. *coli* BL21 (DE3) as inclusion bodies, which were isolated by cell lysis and washing with 0.5% Triton X-100 in 50 mM Tris (pH 8.0) containing 1 mM EDTA, 100 mM NaCl, and 1 mM DTT. Washed inclusion bodies were solubilized in 7 M guanidine hydrochloride, 20 mm Sodium acetate (pH 4.2), and 10 mM DTT. pHLA complexes were assembled by co-refolding HLA-A∗02:01 heavy chain, β2m, and synthetic peptides RMFPNAPYL (RL9) or YMFPNAPYL (YL9) (>95% purity; GL Biochem) at a 1:1:3 M ratio in a redox-controlled buffer containing 100 mM Tris (pH 8.0), 400 mM L-arginine-HCl, 2 mM EDTA, 5 mM reduced glutathione (GSH), and 0.5 mM oxidized glutathione (GSSG). After 48 h at 4 °C, the refolding mixture was dialyzed against 20 volumes of 10 mM Tris-HCl (pH 8.0) for 12 h. The dialysate was purified by anion-exchange chromatography (Q Sepharose, Cytiva), followed by high-resolution Mono Q (Cytiva) and size-exclusion chromatography (Superdex 200 Increase 10/300 Gl, Cytiva).

Recombinant C4 and α7β2 TCR was produced as described previously (13, 35). Briefly, the α and β extracellular domains of the C4 TCR were codon-optimized for *E*. *coli* expression, cloned separately into pET-28a(+) vectors, and expressed in *E*. *coli* BL21 (DE3) as inclusion bodies. Inclusion bodies were isolated and solubilized under the same conditions as for HLA proteins. The α and β chains were co-refolded at a 1:1 M ratio in a redox-controlled buffer containing 100 mM Tris (pH 8.0), 400 mM L-arginine-HCl, 2 mM EDTA, 5 mM reduced glutathione, 0.5 mM oxidized glutathione, and 5 M urea. The refolding mixture was dialyzed sequentially against 20 volumes of 10 mM Tris-HCl (pH 8.0) containing 0.1 M urea for 12 h, and then 20 volumes of 10 mM Tris-HCl (pH 8.0) for another 12 h. Subsequent purification was performed using the same procedures as described for the pHLA proteins.

### Crystallization

The C4 TCR was mixed with peptide-HLA-A∗02:01 complexes at a 1:1 M ratio and concentrated to 10 to 15 mg/mL. Crystallization was performed using the sitting-drop vapor diffusion method at 4 °C. The C4-WT1_126_-_134_-HLA-A∗02:01 complex crystallized in a solution containing 0.1 M ammonium sulfate, 0.1 M sodium citrate, 12% PEG 4000, and 18% glycerol. The C4-YL9-HLA-A∗02:01 complex formed crystals under conditions of 0.1 M magnesium chloride, 0.1 M MES buffer (pH 5.6), and 5% PEG 6000. Crystals were cryoprotected in mother liquor supplemented with 20% (v/v) glycerol and flash-cooled in liquid nitrogen.

### X-ray data collection, processing, and refinement

X-ray diffraction data were collected at 100 K on beamline BL18U1 at the Shanghai Synchrotron Radiation Facility (SSRF). Data were processed using iMosflm and scaled with Aimless from the CCP4 suite (36, 37). Structures were solved by molecular replacement using Phaser (38), with the C4 TCR structure predicted *via* the Swiss-Model server and the WT1_126_-_134_-HLA-A∗02:01 coordinates from PDB entry 3HPJ used as independent search models. Model building was performed in Crystallographic Object-Oriented Toolkit (Coot), and refinement was carried out using Phenix.refine (39, 40).

### Structural analysis and interface calculations

Structural superpositions, docking-orientation inspection, contact analysis, and figure preparation were performed using PyMOL (The PyMOL Molecular Graphics System, Version 3.0.3, Schrödinger, LLC) and UCSF ChimeraX (41). Buried surface area and solvent-accessible surface area were calculated using PDBePISA (42). TCR crossing angles were calculated using the TCR3d TCR angles tool (43, 44). Direct interatomic contacts at the TCR–pHLA interface were assigned and grouped by the contacted peptide/HLA region or TCR CDR loop. H-bonds, SBs, and vdW contacts were identified by structural inspection in PyMOL/ChimeraX and verified manually. H-bonds were assigned using a donor–acceptor distance cutoff of ≤3.5 Å, SBs were assigned between oppositely charged atoms within ≤4.0 Å, and vdW contacts were assigned between nonpolar heavy atoms within ≤4.0 Å. For the contact-distribution analysis shown in Figure 2, *A* and *B*, the percentage for each group was calculated as the number of assigned contacts in that group divided by the total number of assigned direct TCR–pHLA contacts.

### Surface plasmon resonance (SPR)

SPR experiments were performed as described previously, with minor modifications (13). All measurements were carried out on a Biacore 1K instrument (Cytiva) at 25 °C using a CM5 sensor chip. Streptavidin (SA) was covalently immobilized onto both flow cells by standard amine-coupling chemistry, reaching ∼2000 RU. Subsequently, biotinylated C4 TCR was captured onto the experimental flow cell at ∼1000 RU, while a biotinylated, unrelated TCR was immobilized in the reference flow cell under identical conditions as the control for nonspecific binding and bulk refractive index changes. Peptide-HLA complexes (RL9-HLA-A∗02:01 or YL9-HLA-A∗02:01) were injected over both flow cells in HBS-EP + running buffer (10 mM HEPES, pH 7.4, 150 mM NaCl, 3 mM EDTA, 0.005% P20). For each complex, independent serial dilution series in the low-to-high micromolar range were used to ensure well-resolved association and dissociation phases without mass-transport limitation. Each injection included an 80 s association and 300 s dissociation phase. Data were processed and globally fit using Biacore Insight Evaluation Software. For interactions showing monophasic association and dissociation behavior, kinetic parameters were obtained using a 1:1 Langmuir binding model. For datasets showing biphasic association/dissociation behavior, a two-state reaction model was used, in which a single TCR–pHLA binding interaction is described as TCR + pHLA ⇌ TCR–pHLA ⇌ TCR–pHLA∗. For very weak interactions that did not support reliable kinetic fitting, only a conservative affinity estimate was reported. All SPR experiments were performed in three independent replicate measurements, and fitted *K*_D_ values are reported as mean ± SD where reliable fitting was possible.

### Differential scanning fluorimetry (DSF)

DSF was performed as described previously, with minor modifications (33, 45). The thermostability of RL9-HLA-A∗02:01 and YL9-HLA-A∗02:01 was assessed using SYPRO Orange-based DSF on a LightCycler 480 II instrument (Roche). Each 20 μL reaction contained 1 μM protein complex and 1 × SYPRO Orange (Invitrogen) in buffer (10 mM HEPES pH 7.4, 150 mM NaCl, 3 mM EDTA, 0.005% Tween-20). Samples were heated from 25 °C to 95 °C at a ramp rate of 1 °C/min. Fluorescence was recorded using the 533/580 nm excitation/emission filter set. The melting temperature (*T*_m_) was defined as the temperature at which 50% of the protein was unfolded. Experiments were performed in triplicate.

## Data availability

Atomic coordinates and structure factors for C4 TCR–RL9–HLA-A∗02:01 and C4 TCR–YL9–HLA-A∗02:01 have been deposited in the Protein Data Bank (PDB) and are available *via* the RCSB portal at https://www.rcsb.org under accession codes 9WC2 and 9WC3, respectively.

## Supporting information

This article contains supporting information.

## Conflict of interest

The authors declare that they have no conflicts of interest with the contents of this article.
