# Supplementary material for: Enhanced HLA stability of a heteroclitic WT1 vaccine exacts a kinetic penalty in recognition by a natural TCR
Source: J Biol Chem. 2026 Jul 28;302(9):113373. doi: 10.1016/j.jbc.2026.113373 (PMC13524554; doi:10.1016/j.jbc.2026.113373)
Supplement: Supplementary file 1 [file mmc1.pdf]

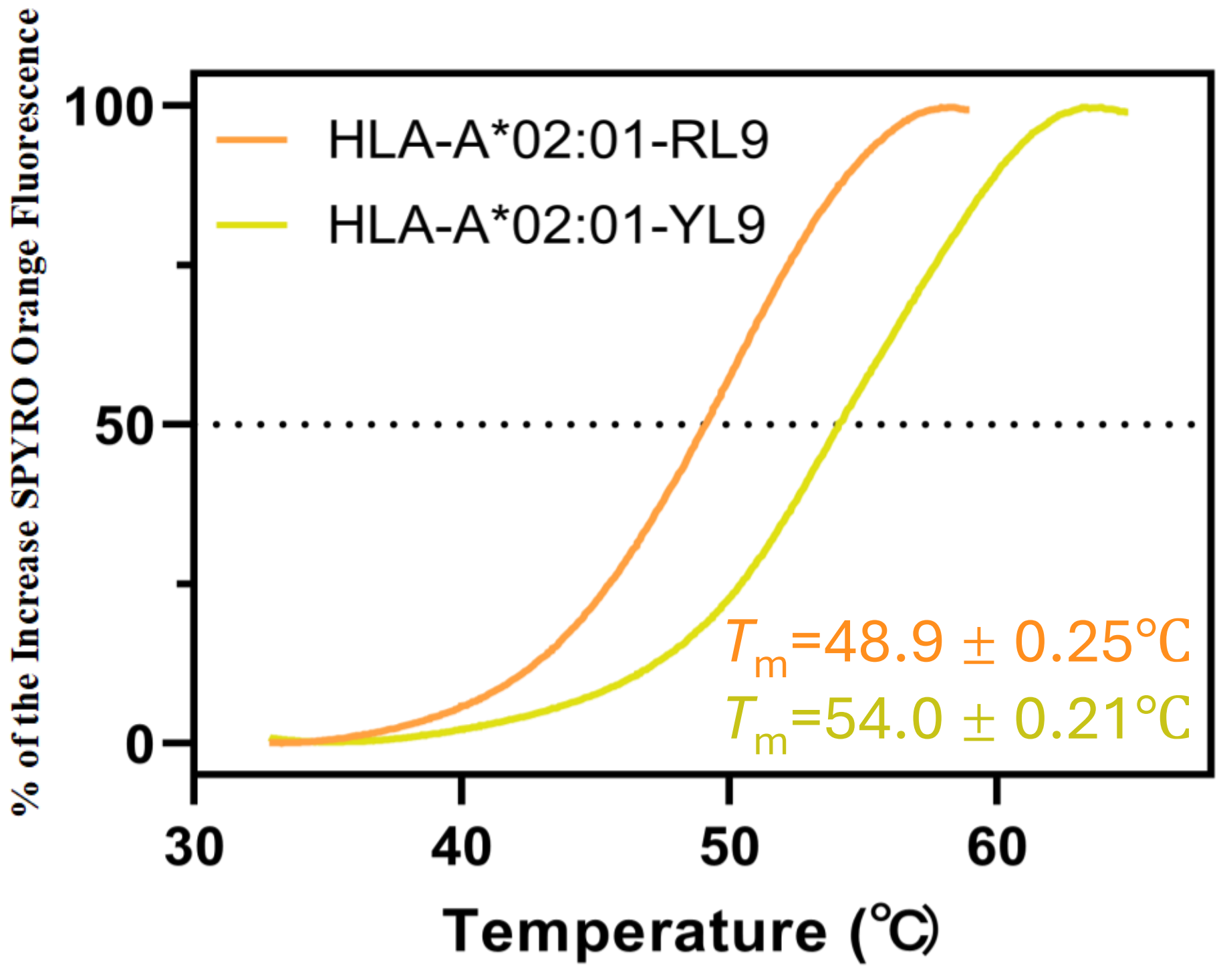

Fig. S1. Thermal stability of RL9–HLA-A*02:01 and YL9–HLA-A*02:01 complexes measured by differential scanning fluorimetry.

Purified RL9–HLA-A*02:01 and YL9–HLA-A*02:01 complexes were subjected to SYPRO Orange-based DSF. Representative melting curves from replicate measurements are shown. RL9–HLA-A*02:01 showed a T_m of 48.9 ± 0.25°C, whereas YL9–HLA-A*02:01 showed a T_m of 54.0 ± 0.21°C, indicating enhanced thermal stability conferred by the R1Y substitution. Data represent mean ± S.D. from three replicate measurements.
