# Supplementary material for: Enhanced HLA stability of a heteroclitic WT1 vaccine exacts a kinetic penalty in recognition by a natural TCR
Source: J Biol Chem. 2026 Jul 28;302(9):113373. doi: 10.1016/j.jbc.2026.113373 (PMC13524554; doi:10.1016/j.jbc.2026.113373)
Supplement: Supplementary file 2 [file mmc2.docx]

**Table S1 Direct contacts of RL9 with different TCRs and TCR-mimic antibodies.**

|  | p1R | p2M | p3F | p4P | p5N | p6A | p7P | p8Y | p9L | Total |
| --- | --- | --- | --- | --- | --- | --- | --- | --- | --- | --- |
| C4 TCR | 3 | 0 | 1 | 6 | 13 | 0 | 0 | 5 | 0 | 28 |
| ESK1 TCRm | 88 | 0 | 0 | 35 | 1 | 0 | 0 | 0 | 0 | 124 |
| 11D06 TCRm | 8 | 0 | 0 | 6 | 9 | 0 | 0 | 2 | 0 | 25 |
| α7β2 TCR | 9 | 0 | 0 | 11 | 9 | 0 | 5 | 39 | 0 | 73 |

A 4 Å cut-off was used for all interactions.

**Table S2 Contact distribution at the C4 TCR–RL9–HLA-A*02:01 interface**

|  | RL9 | | HLAα1 | | HLAα2 | | Total | |  |
| --- | --- | --- | --- | --- | --- | --- | --- | --- | --- |
|  | vdW | H-bond/SB | vdW | H-bond/SB | vdW | H-bond/SB |  |  | |
| TCR CDRα1 | 0 | 0 | 0 | 0 | 14 | 3 | 17 |  |  |
| TCR CDRα2 | 0 | 0 | 0 | 0 | 15 | 0 | 15 |  |  |
| TCR CDRα3 | 6 | 3 | 7 | 2 | 1 | 0 | 19 |  |  |
| TCR CDRβ1 | 4 | 1 | 0 | 0 | 0 | 0 | 5 |  |  |
| TCR CDRβ2 | 0 | 0 | 18 | 2 | 0 | 0 | 20 |  |  |
| TCR CDRβ3 | 12 | 2 | 0 | 0 | 21 | 4 | 39 |  |  |
| Other | 0 | 0 | 0 | 0 | 0 | 1 | 1 |  |  |
| Total | 22 | 6 | 23 | 6 | 51 | 8 | 116 |  |  |

H-bond =hydrogen bond, SB=salt bridge, vdW=van der Waals contact.

A 3.5 Å cut-off was used for H-bonds, and a 4 Å cut-off was used for SBs and vdW.

**Table S3 Residue-level contact profile of RL9 with the C4 TCR and HLA-A*02:01**

|  | p1R | p2M | p3F | p4P | p5N | p6A | p7P | p8Y | p9L | Total |
| --- | --- | --- | --- | --- | --- | --- | --- | --- | --- | --- |
| C4 TCR | 1, 0, 2^#^ | 0, 0, 0 | 1, 0, 0 | 6, 0, 0 | 10, 3, 0 | 0, 0, 0 | 0, 0, 0 | 4, 1, 0 | 0, 0, 0 | 22, 4, 2 |
| HLA-A*02:01 | 39, 3, 1 | 25, 3, 0 | 22, 1, 0 | 3, 0, 0 | 7, 2, 0 | 10, 0, 0 | 7, 0, 0 | 13, 1, 0 | 26, 4, 0 | 152, 14, 1 |

^#^: Values are presented in the order of van der Waals contacts, hydrogen bonds, and salt bridges.

A 3.5 Å cut-off was used for H-bonds, and a 4 Å cut-off was used for SBs and vdW.

**Table S4 C4 TCR-HLA-A*02:01-YL9 contact table**

|  | YL9 | | HLAα1 | | HLAα2 | | Total | |  |
| --- | --- | --- | --- | --- | --- | --- | --- | --- | --- |
|  | vdW | H-bond/SB | vdW | H-bond/SB | vdW | H-bond/SB |  |  | |
| TCR CDRα1 | 0 | 0 | 0 | 0 | 15 | 3 | 18 |  |  |
| TCR CDRα2 | 0 | 0 | 0 | 0 | 10 | 1 | 11 |  |  |
| TCR CDRα3 | 10 | 0 | 8 | 3 | 6 | 1 | 28 |  |  |
| TCR CDRβ1 | 4 | 1 | 0 | 0 | 0 | 0 | 5 |  |  |
| TCR CDRβ2 | 0 | 0 | 16 | 2 | 0 | 0 | 18 |  |  |
| TCR CDRβ3 | 12 | 2 | 0 | 0 | 17 | 4 | 35 |  |  |
| Total | 26 | 3 | 22 | 7 | 48 | 9 | 115 |  |  |

H-bond=hydrogen bond, SB=salt bridge, vdW=van der Waals interactions.

A 3.5 Å cut-off was used for H-bonds, and a 4 Å cut-off was used for SBs and vdW.

**Table S5 Recognition of YL9 by TCR and HLA**

|  | p1Y | p2M | p3F | p4P | p5N | p6A | p7P | p8Y | p9L |  |
| --- | --- | --- | --- | --- | --- | --- | --- | --- | --- | --- |
| C4 TCR | 4, 0, 0^#^ | 0, 0, 0 | 1, 0, 0 | 6, 0, 0 | 11, 2, 0 | 0, 0, 0 | 0, 0, 0 | 4, 1, 0 | 0, 0, 0 | 26, 3, 0 |
| HLA-A*02:01 | 40, 3, 0 | 31, 3, 0 | 23, 2, 0 | 4, 0, 0 | 3, 2, 0 | 8, 0, 0 | 8, 0, 0 | 13, 1, 0 | 27, 4, 0 | 157, 15, 0 |

^#^: Values are presented in the order of van der Waals contacts, hydrogen bonds, and salt bridges.

A 3.5 Å cut-off was used for H-bonds, and a 4 Å cut-off was used for SBs and vdW.

**Table S6. Thermal stability of pHLA-A*02:01.**

| *T*m (℃) | Repeat 1 | Repeat 2 | Repeat 3 | Mean ± S.D. |
| --- | --- | --- | --- | --- |
| HLA-A*02:01-RL9 | 49.1 | 48.9 | 48.6 | 48.9 ± 0.25 |
| HLA-A*02:01-YL9 | 54.1 | 54.2 | 53.8 | 54.0 ± 0.21 |

S.D., standard deviation.

**Table S7. Binding affinity of pHLA-A*02:01 to C4 TCR.**

|  | *k*_a1_ (1/M·s) | *k_d_*_1_ (1/s) | *k*_a2_ (1/s) | *k*_d2_ (1/s) | *K*_D_ (μM) |
| --- | --- | --- | --- | --- | --- |
| HLA-A*02:01-RL9 | (9.69 ± 1.03)  *10^4^ | 1.51 ± 0.07 | (2.47 ± 0.41)  *10^-3^ | (2.08 ± 0.54)  *10^-2^ | 13.8 ± 1.53 |
| HLA-A*02:01-YL9 |  |  |  |  | >150 |

Binding affinities (*K*_D_) were determined by SPR.

Reliable kinetic fitting was not obtained for the YL9–HLA-A*02:01 interaction; therefore, the affinity is reported conservatively as KD >150 μM.

**Table S8. Binding affinity of pHLA-A*02:01 to α7β2 TCR.**

|  | *k*_a1_ (1/M·s) | | *k_d_*_1_ (1/s) | | *k*_a2_ (1/s) | | *k*_d2_ (1/s) | | *K*_D_ |
| --- | --- | --- | --- | --- | --- | --- | --- | --- | --- |
| HLA-A*02:01-RL9 | (5.54 ± 0.33)  *10^5^ | (2.16 ±0.197)  *10^-2^ | |  | |  | | 38.77 ± 6.24 nM | |
| HLA-A*02:01-YL9 | (1.28 ±0.105)  *10^5^ | (8.09 ±0.285)  *10^-1^ | | (3.01 ±0.046)  *10^-3^ | | (1.78 ± 0.15)  *10^-2^ | | 5.42 ± 0.59 μM | |

Binding affinities (*K*_D_) were determined by SPR.
